# Supplementary material for: A systematic review of machine learning approaches for MRI-based brain tumor analysis
Source: Front Comput Neurosci. 2026 Oct 12;20:1937002. doi: 10.3389/fncom.2026.1937002 (PMC13696301; doi:10.3389/fncom.2026.1937002)
Supplement: Supplementary file 1 [file Data_Sheet_1.zip › Submission_Upload/main_frontiers.pdf]

A Systematic Review of Machine Learning Approaches for MRI-Based Brain Tumor Analysis

Ivan Viktorovich Brak [1,2], Merich Doruk Kaymakcioglu [1], Vasiliy Vladimirovich Belousov [3], Zhanibek Baiturlin [4], Daultay Azamatovich Batyrkhanov [4], Daniil Olegovich Mityagin [2], Manzura Zholdassova [6] and Almira Melsovna Kustubayeva [5,6,*]

[1] *Department of Mathematical Modeling and Artificial Intelligence, Institute of Computer Science and Telecommunications, Peoples' Friendship University of Russia (RUDN University), Moscow, Russia*
[2] *Department of Automated Control Systems, National University of Science and Technology MISIS, Moscow, Russia*
[3] *Federal Research Center "Computer Science and Control" of the Russian Academy of Sciences (FRC CSC RAS), Moscow, Russia*
[4] *Department of Radiology and Radiosurgery, JSC "National Center for Neurosurgery", Astana, Kazakhstan*
[5] *Department of Biophysics, Biomedicine and Neuroscience, Al-Farabi Kazakh National University, Almaty, Kazakhstan*
[6] *Brain Institute, Al-Farabi Kazakh National University, Almaty, Kazakhstan**[1] Department of Mathematical Modeling and Artificial Intelligence, Institute of Computer Science and Telecommunications, Peoples' Friendship University of Russia (RUDN University), Moscow, Russia*
[2] *Department of Automated Control Systems, National University of Science and Technology MISIS, Moscow, Russia*
[3] *Federal Research Center "Computer Science and Control" of the Russian Academy of Sciences (FRC CSC RAS), Moscow, Russia*
[4] *Department of Radiology and Radiosurgery, JSC "National Center for Neurosurgery", Astana, Kazakhstan*
[5] *Department of Biophysics, Biomedicine and Neuroscience, Al-Farabi Kazakh National University, Almaty, Kazakhstan*
[6] *Brain Institute, Al-Farabi Kazakh National University, Almaty, Kazakhstan*

Correspondence*:
Almira Melsovna Kustubayeva, almira.Kustubaeva@kaznu.kzAlmira Melsovna Kustubayeva
almira.Kustubaeva@kaznu.kz

ABSTRACT

Machine learning is now widely applied to MRI-based brain tumor analysis, with task-specific performance metrics: the Dice Similarity Coefficient (DSC) quantifies segmentation overlap, while AUC and accuracy measure classification performance. We conducted a PRISMA 2020 systematic review, searching Scopus, Web of Science, PubMed, and IEEE Xplore, and identified 213 eligible studies published between January 2018 and the final search date of 16 March 2026. Segmentation performance was synthesized by random-effects meta-analysis and classification performance was summarized descriptively. Classification, detection, and grading tasks dominated the literature, followed by segmentation; U-Net variants were the most frequent architectures, with Transformer-based models growing fastest. The pooled mean DSC was 0.871 (95% CI: 0.852–0.889) with a 95% prediction interval of 0.788–0.953, and heterogeneity was extreme (I^2 = 99.9%), so the pooled mean should be read as a descriptive summary of the literature rather than a single underlying effect. The descriptive mean AUC across classification studies was 0.960. Methodological gaps were pervasive: only 18.8% of studies incorporated explainability, only 18.8% performed external validation, and none reported robustness metrics. Benchmark accuracy has outpaced clinical evidence: until models are validated on independent cohorts, evaluated for robustness under realistic acquisition shifts, and made interpretable to clinicians, reported performance is unlikely to translate into patient care.

Keywords: brain tumor segmentation, brain tumor classification, machine learning, deep learning, systematic review, meta-analysis, MRI, Dice similarity coefficient

1 INTRODUCTION

1.1 Clinical Significance of Brain Tumors

Brain tumors represent one of the most clinically and biologically challenging groups of central nervous system (CNS) neoplasms (1). Globally, brain and other CNS cancers account for approximately 321,000 new cases and 248,000 deaths annually, ranking among the top causes of cancer-related disability-adjusted life years (2). In the United States alone, the Central Brain Tumor Registry (CBTRUS) reports an annual incidence exceeding 100,000 primary brain and other CNS tumors (3). Glioblastoma (GBM, WHO grade IV) accounts for approximately 50% of all malignant primary brain tumors (3), with a poor prognosis: median overall survival of 14.6–16.0 months with standard chemoradiotherapy and a five-year survival rate below 7% (4). The 2021 WHO Classification of Tumors of the Central Nervous System introduced over 200 histologically and molecularly defined tumor entities, a sign of the heterogeneity of these neoplasms and the consequent demand for precise diagnostic and monitoring tools (1).

Accurate delineation of tumor boundaries (distinguishing enhancing tumor from surrounding healthy parenchyma, peritumoral edema, and necrotic core) is fundamental to multiple aspects of clinical care. It underpins neurosurgical planning (maximizing extent of resection while preserving eloquent cortex), radiation therapy targeting (defining gross tumor volume and clinical target volume margins), and longitudinal treatment response assessment according to the Response Assessment in Neuro-Oncology (RANO) criteria. Errors in boundary delineation directly translate into suboptimal treatment or unnecessary tissue damage, both of which adversely affect patient survival and quality of life.

1.2 Role of MRI in Brain Tumor Imaging

Magnetic resonance imaging (MRI) is the gold-standard modality for brain tumor visualization, offering superior soft-tissue contrast without ionizing radiation (5). Clinical brain tumor protocols typically acquire multiple complementary pulse sequences, each highlighting different tissue characteristics:

- **T1-weighted (T1):** delineates anatomical structure and identifies regions of hemorrhage or calcification;
- **T1 contrast-enhanced (T1ce):** gadolinium-based contrast agents enhance regions of blood–brain barrier breakdown, highlighting the active enhancing tumor core;
- **T2-weighted (T2):** provides sensitivity to water content, which helps visualize peritumoral edema and non-enhancing tumor infiltration;
- **Fluid-Attenuated Inversion Recovery (FLAIR):** suppresses cerebrospinal fluid signal while retaining sensitivity to periventricular and cortical lesions.

The complementary information provided by these sequences enables clinicians to identify distinct tumor sub-compartments, yet integrating multi-modal information remains cognitively demanding and introduces subjectivity into the interpretation process.

1.3 Challenges of Manual Segmentation

Manual segmentation performed by experienced neuroradiologists remains the clinical reference standard for tumor boundary delineation. However, it suffers from several well-documented limitations. It is very time-consuming: expert delineation of a single multi-sequence brain MRI study may require 20–60 minutes or more, depending on tumor complexity and the number of sub-regions annotated (5). Manual segmentation also exhibits substantial inter-observer variability, with reported Dice overlap between expert raters often ranging from 74% to 85% for the whole tumor region, and considerably lower for ambiguous sub-compartments such as the non-enhancing tumor core. Intra-observer variability compounds reproducibility concerns further, with repeated delineations by the same expert showing variation of approximately 20%. On top of this, the steadily growing volume of neuroimaging examinations (driven by expanded screening indications, longitudinal monitoring protocols, and clinical trials) has created a workload that far exceeds available radiologist capacity. Together, these limitations point to a clear clinical need for automated, reproducible, and scalable approaches to brain tumor boundary delineation.

1.4 Evolution of Machine Learning Approaches

Machine learning (ML) is now the primary computational approach for automating brain tumor analysis in MRI. Early approaches relied on traditional ML algorithms such as support vector machines (SVMs), random forests, and conditional random fields, combined with hand-engineered features derived from intensity histograms, texture descriptors, and spatial priors. While these methods achieved moderate accuracy, they required extensive manual feature engineering and generalized poorly across institutions and acquisition protocols.

Deep learning reshaped the field. Convolutional neural networks (CNNs) (6) introduced the ability to learn hierarchical feature representations directly from raw voxel data, eliminating the feature engineering bottleneck. The U-Net architecture (7), with its symmetric encoder–decoder structure and skip connections, became the foundational model for biomedical image segmentation due to its ability to produce dense pixel-wise predictions while preserving spatial detail. Subsequent architectural innovations, including residual connections (ResNet) (8), dense connections (DenseNet), and attention mechanisms, further

improved segmentation performance by addressing gradient degradation and allowing selective feature emphasis.

More recently, Vision Transformers (9) and their variants (Swin Transformer, SegFormer) have introduced global self-attention mechanisms (10) that capture long-range spatial dependencies, which convolutional architectures model only indirectly through receptive field expansion. Hybrid CNN–Transformer models that combine the local inductive biases of convolutions with the global context modeling of self-attention are the latest development, achieving the best results on major benchmarks.

The Brain Tumor Segmentation (BraTS) challenge series (5, 11), running annually since 2012, has driven progress by providing standardized training and evaluation data with expert annotations. The Dice Similarity Coefficient (DSC) (12) has become the dominant evaluation metric for segmentation tasks, with top-performing models now exceeding 0.90 DSC on enhanced tumor, whole tumor, and tumor core sub-regions. The nnU-Net framework (13) showed that systematic optimization of preprocessing pipelines, training protocols, and post-processing steps can match or exceed hand-tuned architectures, showing that the entire ML pipeline matters more than architecture alone.

1.5 Limitations of Prior Reviews

Several systematic and narrative reviews of ML for brain tumor imaging have been published in recent years (Table 1). Most focus on a single task such as segmentation or classification (14), cover periods that predate the architectural shifts of 2023–2026, or summarize the literature descriptively without quantitative synthesis (15, 16, 17). The only prior meta-analysis, by Wang et al. (18), pooled 19 segmentation studies with external validation and did not address classification, radiogenomics, or robustness. Three dimensions of methodological quality (explainability, external validation, and robustness to domain shift) remain largely unassessed across the field. This review addresses these gaps by combining a DSC-focused quantitative synthesis of segmentation performance with a structured audit of explainability, external validation, and robustness reporting across all major MRI-based brain tumor analysis tasks. Because segmentation overlap metrics and classification discrimination metrics answer different clinical and statistical questions, the two endpoint families are analyzed separately throughout.

Table 1. Comparison of this review with recent related systematic reviews and meta-analyses of ML for brain tumor MRI analysis.

Review	Scope	*N*	Period	Meta-analysis	RoB tool	XAI	ExtVal	Robustness
Wang et al. (18)	Seg. detection	19	2015–2023	Yes (DSC)	QUADAS-2, CLAIM	No	Yes	No
Satushe et al. (15)	Diagnosis (ML/DL)	155	2010–2025	No	No	Partial	No	No
Pasunoori et al. (14)	Seg. detection	NR	NR	No	No	No	No	No
Fahim et al. (16)	Det. class. seg.	NR	NR	No	No	Partial	No	No
Dorfner et al. (17)	Seg. class. prog.	NR	NR	No (narrative)	No	Partial	Partial	No
**This review**	**All tasks**	**213**	**2018–Mar 2026**	**Yes (DSC)**	**Custom + mapping**	**Yes**	**Yes**	**Yes**

1.6 Objectives of This Review

To address the gaps identified above, we conducted a systematic review following the Preferred Reporting Items for Systematic Reviews and Meta-Analyses (PRISMA) 2020 guidelines (19). The review covers studies published from 2018 onward because this marks the point at which end-to-end deep learning became the dominant paradigm in brain tumor MRI analysis, following the standardization of the BraTS benchmark

ecosystem and the widespread adoption of encoder–decoder architectures; earlier feature-engineering pipelines are already covered by previous reviews.

Our specific objectives are to:

1. characterize the distribution of ML architectures, tasks, datasets, and evaluation practices across the full breadth of tasks that prior single-task reviews have not covered together;
2. address the absence of quantitative synthesis in most prior reviews by performing a random-effects meta-analysis of segmentation performance using the DSC, with heterogeneity assessment and prediction intervals;
3. assess methodological quality with respect to the three dimensions left unexamined by prior reviews: explainability (XAI), external validation, and robustness testing;
4. translate the identified gaps into concrete, actionable recommendations for future research and clinical translation.

Section 2 describes the methods, Section 3 the results, Section 4 discusses implications and limitations, and Section 5 concludes.

2 MATERIALS AND METHODS

This systematic review was conducted in accordance with the Preferred Reporting Items for Systematic Reviews and Meta-Analyses (PRISMA) 2020 guidelines (19). The completed PRISMA 2020 checklist is provided in Supplementary File S1. The review protocol was retrospectively registered on the Open Science Framework (OSF) on 19 May 2026 (registration DOI: 10.17605/OSF.IO/H4YF7); the search had been initiated prior to registration, which is noted as a limitation.

2.1 Search Strategy

We searched four electronic databases: Scopus, Web of Science, PubMed, and IEEE Xplore. Searches were not restricted at the database-query stage; eligibility was subsequently limited to studies published from 1 January 2018 to the final search date of 16 March 2026. The search strategy combined terms related to three conceptual domains using Boolean operators:

- **Domain 1 (Condition):** “brain tumor” OR “brain tumour” OR “glioma” OR “glioblastoma” OR “meningioma” OR “brain metastasis” OR “brain neoplasm”
- **Domain 2 (Imaging):** “MRI” OR “magnetic resonance imaging” OR “neuroimaging”
- **Domain 3 (Method):** “machine learning” OR “deep learning” OR “neural network” OR “segmentation” OR “classification” OR “convolutional neural network” OR “U-Net” OR “transformer”

The final query was constructed as: (Domain 1) AND (Domain 2) AND (Domain 3). Table 2 reports the exact query syntax, search date, and number of records retrieved for each database. Duplicates were identified and removed using a combination of automated DOI matching and manual title–author comparison, yielding 1,535 unique records after removal of 312 duplicates. Reference lists of included studies and relevant review articles were hand-searched to identify additional eligible publications.

Table 2. Search strategy details for each electronic database. Searches were initially run in January 2024 and updated periodically; the date shown is that of the final search, which defines the review cutoff.

**Database**	**Search date**	**Records**	**Fields searched**
Scopus	16 March 2026	687	Title, Abstract, Keywords
Web of Science	16 March 2026	524	Topic (Title, Abstract, Author Keywords, Keywords Plus)
PubMed	16 March 2026	412	Title, Abstract, MeSH Terms
IEEE Xplore	16 March 2026	224	Full text and metadata
**Total**		**1,847**	
After deduplication		1,535	

2.2 Eligibility Criteria

Studies were included if they met all of the following criteria: (1) used human brain tumor MRI data (any field strength, any sequence combination); (2) applied one or more ML or deep learning methods; (3) addressed at least one of the following tasks: segmentation, classification, detection, grading, prognosis, synthesis, or radiogenomics; (4) reported quantitative evaluation metrics (for segmentation: DSC, HD95, or volumetric overlap; for classification/detection: accuracy, AUC, sensitivity, specificity, or precision/recall); (5) were published as peer-reviewed journal articles or conference proceedings in English; and (6) were published between 1 January 2018 and 16 March 2026.

Studies were excluded if they: (1) were review articles, editorials, commentaries, or preprints without original experimental results; (2) did not use MRI as the primary imaging modality; (3) focused exclusively on non-tumor brain pathologies (e.g., stroke, multiple sclerosis) or on non-human data; (4) reported only qualitative or visual results without quantitative metrics; (5) were duplicate publications; or (6) had a sample size of fewer than 10 patients, to avoid extremely underpowered feasibility reports.

2.3 Study Selection

Study selection followed a two-stage, independent parallel screening process. In Stage 1 (title and abstract screening), two reviewers independently screened all retrieved records against the eligibility criteria. In Stage 2 (full-text screening), the same two reviewers independently assessed full texts of records that passed Stage 1. Disagreements at either stage were resolved through discussion; if no consensus was reached, a third reviewer acted as arbiter. Inter-rater agreement was assessed using Cohen's κ ($\kappa = 0.87$ for title/abstract screening, $\kappa = 0.92$ for full-text screening). The selection process yielded 213 studies for inclusion in the final review.

2.4 Data Extraction

A standardized data extraction form comprising 88 variables was developed to capture detailed information from each included study. The extraction template was organized into the following categories:

- **Study identification:** Paper ID, authors, year, title, journal, DOI, country, study design.
- **Data characteristics:** Dataset name, sample size, tumor type, MRI modalities, number of modalities.
- **Tumor feature fields:** Morphological (volume, diameter, sphericity), intensity (contrast enhancement, necrosis ratio), texture (GLCM, GLRLM, LBP, wavelet), and location (lobe, midline shift, lateralization) descriptors.
- **Model characteristics:** Task solved, model type, tested hypothesis.

- **Quality metrics:** DSC (mean and SD), HD95, cross-validation folds, external validation, explainability, inference time.
- **Synthesis metrics:** FID, SSIM, PSNR, Dice improvement.
- **Clinical metrics:** AUC, expected calibration error, physician trust assessment.
- **Biological correlation:** IDH correlation, MGMT correlation, histopathological match.
- **Robustness metrics:** Noise drop, resolution drop, domain shift drop.
- **PRISMA compliance:** Search reported, bias tool used, effect measure, registration, data availability.
- **Risk of bias:** Overall judgment, funding source, conflict of interest, protocol availability.

Data extraction was performed independently by two reviewers, with discrepancies resolved through consultation with a third reviewer. The tumor feature fields (morphological, intensity, texture, and location descriptors) were extracted where available but proved too sparsely and heterogeneously reported across studies to support quantitative analysis; they are retained in the full extraction table (Supplementary File S3) for reuse by other researchers but are not analyzed further in this review.

2.5 Data Normalization and Quality Control

Several data quality issues were identified and addressed during extraction. DSC and AUC values reported on a 0–100 percentage scale were normalized to the 0–1 scale. Categorical variables (explainability, external validation) were standardized to binary coding (0/1). Free-text model type descriptions were categorized into seven high-level groups: U-Net variants, ResNet variants, CNN (other), Transformer-based, Ensemble/Hybrid, GAN-based, and Traditional ML. Task descriptions were categorized as Classification/Detection/Grading, Segmentation, Segmentation+Classification, Prognosis/Survival, Synthesis/Generation, and Radiogenomics.

2.6 Statistical Analysis

2.6.1 Descriptive Statistics

Frequency distributions were computed for categorical variables (model type, task type, dataset, country, explainability, external validation). Continuous metrics (DSC, AUC) were summarized using mean, median, standard deviation, and range.

2.6.2 Heterogeneity Assessment

Statistical heterogeneity was assessed using Cochran's Q statistic and the I^2 index (20). The I^2 index was calculated as:

$$I^2 = \max\left(0, \frac{Q-(k-1)}{Q} \times 100\%\right) \tag{1}$$

where Q is the Cochran's Q statistic and k is the number of studies. A DerSimonian–Laird random-effects model was fitted, with study-level weights incorporating both within-study variance and the estimated between-study variance: $w_i = 1/(\mathrm{SE}_i^2 + \hat{\tau}^2)$, where $\mathrm{SE}_i = \sigma_i/\sqrt{n_i}$ and $\hat{\tau}^2$ is the DerSimonian–Laird estimate of between-study heterogeneity. A 95% prediction interval was computed to characterize the range of plausible true effects in a new study. Leave-one-out sensitivity analysis was performed to assess the influence of individual studies on the pooled estimate. This analysis was restricted to the 15 studies reporting DSC mean, standard deviation, and sample size. These 15 studies constitute the complete set providing the paired mean, variance, and sample-size data required for inverse-variance weighting; k was

therefore determined by reporting completeness in the literature rather than chosen by design. This number exceeds the commonly cited minimum of approximately 10 studies for random-effects pooling and for Egger's regression test, but it limits the statistical power of moderator analyses, which is why all subgroup comparisons are presented as exploratory.

2.6.3 Publication Bias

Publication bias was assessed visually using funnel plots, with standard error ($\mathrm{SE} = \sigma/\sqrt{n}$) plotted against effect size (DSC). Funnel plot asymmetry was tested formally using Egger's regression test, which is recommended when $k \geq 10$ studies are available.

2.6.4 Subgroup Analyses

Subgroup comparisons were performed using the Kruskal–Wallis H test for multi-group comparisons (model type, task type) and the Mann–Whitney U test for two-group comparisons (explainability, external validation). Statistical significance was set at $\alpha = 0.05$.

2.6.5 Regression Analysis

Ordinary least squares (OLS) multiple regression was performed with DSC as the dependent variable and year, sample size, number of MRI modalities, and explainability (binary) as independent variables. Standardized coefficients (β) were computed to assess relative predictor importance.

2.6.6 Risk of Bias in Individual Studies

Because the included studies covered heterogeneous tasks (segmentation, classification, detection, radiogenomics, prognosis, synthesis), no single validated risk-of-bias tool applies meaningfully to all 213 studies; many are not prediction-model studies in the sense assumed by tools such as PROBAST. We therefore used two complementary assessments that answer different questions, rather than two parallel standards of rigor: a field-wide reporting appraisal of every included study, and a formal risk-of-bias assessment of the subset whose results enter the quantitative pooling, where bias directly affects the pooled estimate.

2.6.6.1 Field-wide reporting appraisal (all 213 studies).

A custom 8-item checklist, adapted from CLAIM (21) and PRISMA-AI, assessed: (1) clear inclusion/exclusion criteria, (2) baseline comparison, (3) statistical testing reported, (4) external validation, (5) explainability, (6) data availability, (7) conflict of interest, (8) study design clarity. Each item was scored as "yes", "no", or "unclear". This appraisal characterizes reporting quality across the whole field and feeds the descriptive quality profile in Section 3.7; it is not itself a validated risk-of-bias instrument.

2.6.6.2 Formal risk-of-bias assessment (15 meta-analysis studies).

For the studies contributing to the quantitative meta-analysis, a formal assessment was performed using PROBAST+AI, a 2025 extension of the Prediction model Risk Of Bias ASsessment Tool for AI/ML prediction models (22). Two reviewers independently rated each study on four domains: participants, predictors, outcome, and analysis. Overall risk of bias was judged as "low", "high", or "unclear". Disagreements were resolved by consensus. The PROBAST+AI results are summarized in Supplementary File S4. Studies with high overall bias were not excluded but were investigated in the leave-one-out sensitivity analysis and discussed in the limitations.

2.6.7 Certainty of Evidence

A formal GRADE assessment was not performed due to extreme heterogeneity across task types, target conditions, outcome metrics, and evaluation protocols. Applying GRADE meaningfully would require separate evidence bodies for segmentation, classification/detection, radiogenomics, synthesis, and prognosis, each with task-specific outcomes and risk-of-bias considerations. Instead, we characterize certainty qualitatively using heterogeneity analyses, reporting completeness, external validation status, publication-bias assessment, and predefined methodological quality indicators.

All statistical analyses were performed using Python 3.12 (pandas, NumPy, SciPy, statsmodels, matplotlib, seaborn) and R 4.3 (meta, metafor, dmetar). A two-sided $p < 0.05$ was considered statistically significant unless otherwise stated.

2.6.8 Meta-Analysis of DSC: Effect Size and Transformation

DSC is a proportion bounded between 0 and 1 whose variance is heteroscedastic near the boundaries. To satisfy normality assumptions, the logit transformation was applied before pooling:

$$\mathrm{logit}(\mathrm{DSC}) = \ln\left(\frac{\mathrm{DSC}}{1-\mathrm{DSC}}\right) \tag{2}$$

The within-study variance of the logit-transformed DSC was approximated using the delta method:

$$\mathrm{Var}(\mathrm{logit}(\mathrm{DSC})) = \frac{\mathrm{Var}(\mathrm{DSC})}{[\mathrm{DSC}(1-\mathrm{DSC})]^2} \tag{3}$$

where $\mathrm{Var}(\mathrm{DSC}) = \mathrm{SD}^2$. A DerSimonian–Laird random-effects model was fitted to the logit DSC. The pooled estimate and its confidence and prediction intervals were back-transformed to the original DSC scale using the inverse logit. Sensitivity analyses included: (I) leave-one-out analysis; (II) restricted maximum likelihood (REML) estimator of $\hat{\tau}^2$; (III) Hartung–Knapp adjustment for the confidence interval; and (IV) analysis on the raw (untransformed) DSC scale for comparability with earlier drafts (results in Supplementary File S5).

2.6.9 Handling of Missing Data

For descriptive statistics, missing values were excluded pairwise. For the meta-analysis, only studies with complete DSC mean, standard deviation, and sample size were included (complete-case analysis; 15 of 61 DSC-reporting studies). No imputation was performed because of the small number of missing variance estimates and the risk of introducing bias. As a sensitivity check for the impact of this restriction, the complete-case pooled estimate was compared with the descriptive mean of all 61 DSC-reporting studies (Section 3.5).

3 RESULTS

3.1 Study Selection and Characteristics

The systematic search and screening process yielded 213 studies meeting the inclusion criteria. Figure 1 presents the PRISMA flow diagram summarizing the study selection process.

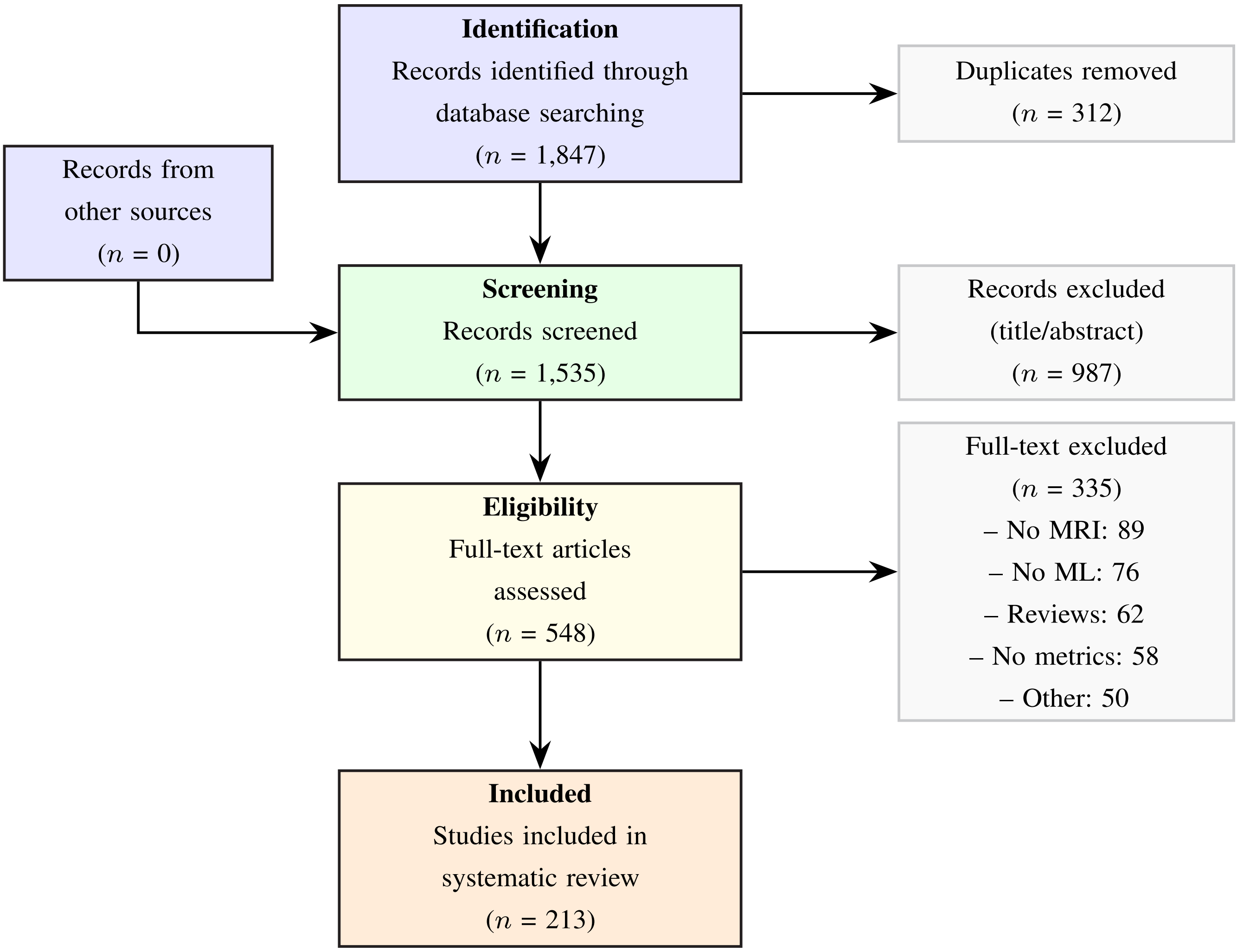

Figure 1. PRISMA flow diagram of the study selection process.

The 213 included studies were published between January 2018 and the 16 March 2026 search cutoff and show near-exponential growth. Publication counts rose from 1 study in 2018 to 82 in 2025, with 30 already published in 2026 by the search cutoff (Figure 2). Output grew rapidly between 2022 and 2025 (16 $\rightarrow$ 22 $\rightarrow$ 58 $\rightarrow$ 82 studies), with the sharpest increase occurring between 2023 and 2024.

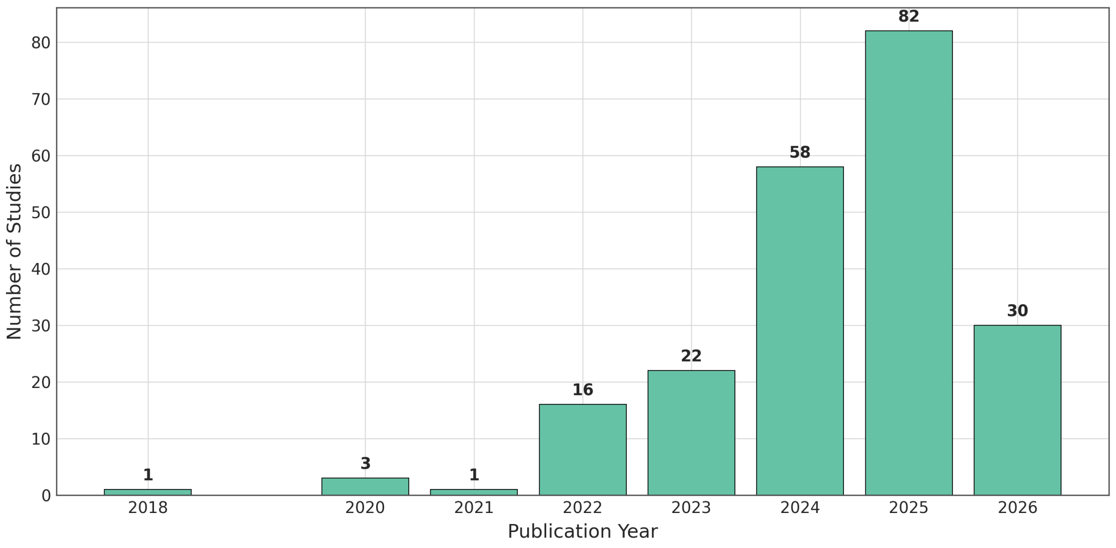

Figure 2. Publication trends by year across 213 included studies (January 2018 to the 16 March 2026 search cutoff).

The geographic distribution revealed a strong concentration in South and East Asia. India contributed the largest share of publications (72/213, 33.8%), followed by China (14, 6.6%), Bangladesh (9, 4.2%), Saudi Arabia (8, 3.8%), and the United States (7, 3.3%). European countries together contributed approximately 15% of studies, with Italy, Spain, and Ireland each contributing 2–4 publications.

3.2 Data Sources and Benchmarks

Figure 3 shows the datasets used across studies. Kaggle-hosted datasets (Br35H, SARTAJ, Nickparvar brain tumor collections) were the most common data sources (approximately 30.5%), followed by BraTS challenge datasets (21.1%), custom institutional datasets (18.8%), Figshare brain tumor datasets (14.1%), and The Cancer Imaging Archive (TCIA; 8.0%). Sixteen studies (7.5%) did not report which dataset they used.

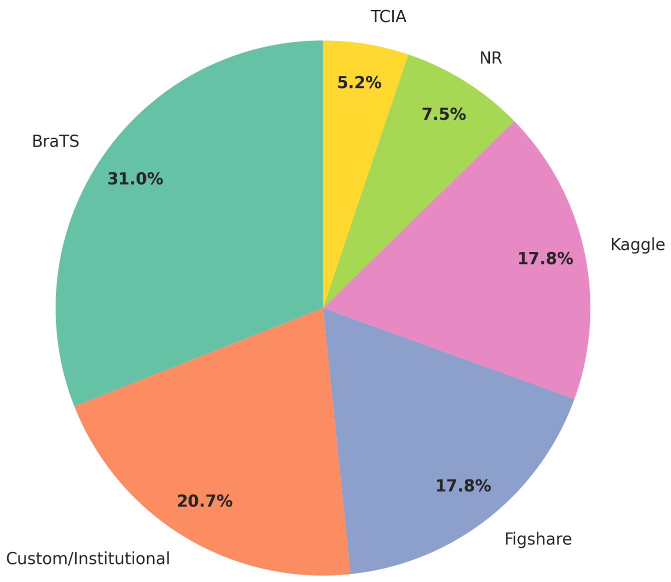

Figure 3. Distribution of data sources across 213 included studies. Kaggle-hosted datasets dominate classification studies, while BraTS challenge datasets are predominantly used for segmentation tasks. TCIA = The Cancer Imaging Archive; NR = Not Reported.

BraTS 2020 was the single most popular individual dataset and appeared overwhelmingly in segmentation work; Kaggle-sourced datasets, by contrast, fed mostly classification studies. Many groups combined several public datasets to enlarge their training sets or to enable external validation. Dataset fragmentation was severe: most unique dataset names appeared in only one or two studies, limiting cross-study comparability.

3.3 Task Distribution

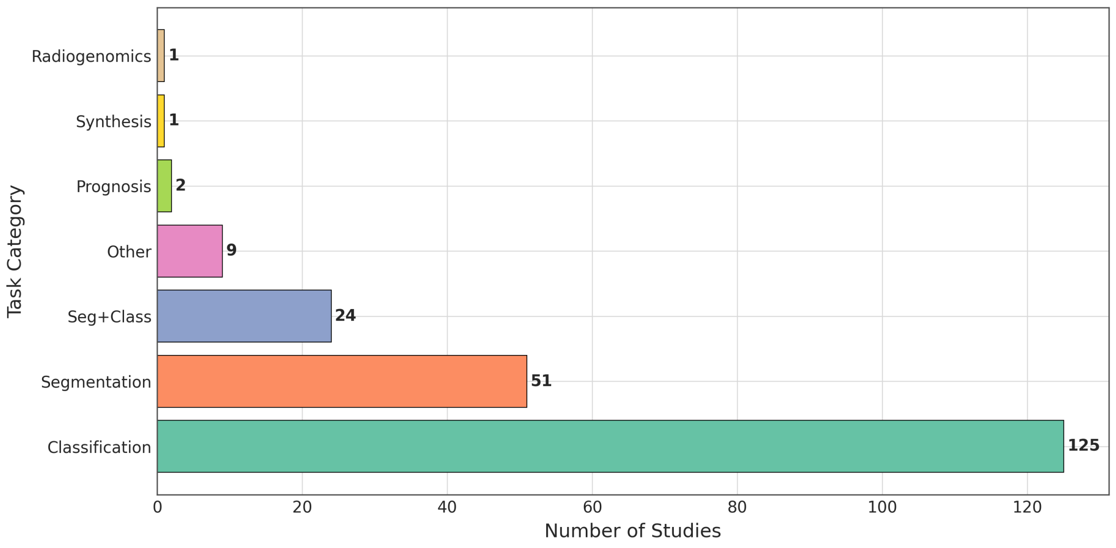

Figure 4. Distribution of ML tasks addressed across 213 included studies. Classification and detection tasks collectively form the largest category, while emerging tasks such as radiogenomics and synthesis remain underrepresented.

Figure 4 and Table 3 summarize the ML tasks addressed. Classification, detection, and grading together formed the largest category (approximately 95 studies, 44.6%), followed by segmentation (75 studies, 35.2%) and joint segmentation-classification workflows (25 studies, 11.7%). Less common tasks included prognosis and survival prediction (3.8%), image synthesis and generation (2.3%), and radiogenomics, i.e., predicting molecular markers such as IDH mutation and MGMT promoter methylation from imaging data (2.3%).

Table 3. Distribution of machine learning tasks across 213 included studies.

Task Category	Count	Share (%)
Classification / Detection / Grading	95	44.6
Segmentation	75	35.2
Segmentation + Classification	25	11.7
Prognosis / Survival Prediction	8	3.8
Synthesis / Generation	5	2.3
Radiogenomics (IDH/MGMT prediction)	5	2.3

3.4 Model Architecture Analysis

Table 4 summarizes the ML architectures used. U-Net and its variants (Modified U-Net, nnU-Net, Attention U-Net, 3D U-Net) were the most common family, appearing in 41 studies (19.2%).

ResNet variants (ResNet50, ResNet101, ResNet152) constituted the second most common category (29 studies, 13.6%), serving both as standalone classifiers and as backbone feature extractors within larger

pipelines (8). Other CNN architectures (custom designs, EfficientNet at 4.2%, VGG variants at 4.2%, DenseNet at 1.9%, and YOLO-based detection models at 2.3%) made up approximately 25% of studies.

Transformer-based architectures, including Vision Transformers (ViT), Swin Transformers, and hybrid CNN-Transformer designs, appeared in 20 studies (9.4%) (9, 10). Although relatively new, Transformers were the fastest-growing category, with adoption concentrated in 2024–2026.

Ensemble and fusion models (3.8%) combined multiple architectures or modalities to improve robustness. GAN-based approaches (1.4%) were primarily used for data augmentation or synthetic image generation rather than direct segmentation. Classical ML methods (SVM, KNN, Random Forest; approximately 10%) persisted but showed a clear declining trend in recent years.

Table 4. Distribution of model architecture categories across 213 included studies. Each study was assigned to one primary category based on its dominant architecture. Nine studies with unspecified or ambiguous model descriptions were categorized as "Unknown" and are excluded from this table.

Architecture Category	Count	Share (%)
U-Net variants	41	19.2
ResNet variants	29	13.6
CNN (other custom)	26	12.2
Transformer / ViT / Swin	20	9.4
SVM / Classical ML	14	6.6
EfficientNet	9	4.2
VGG variants	9	4.2
Ensemble / Fusion	8	3.8
Hybrid models	6	2.8
YOLO (detection-based)	5	2.3
Inception / GoogLeNet	4	1.9
DenseNet	4	1.9
GAN variants	3	1.4
Other / custom	26	12.2
Unknown	9	4.2
**Total**	**213**	**100.0**

3.5 Quantitative Meta-Analysis

3.5.1 Dice Similarity Coefficient Distribution

Of the 213 included studies, 61 (28.6%) reported the Dice Similarity Coefficient as a primary evaluation metric. The distribution of DSC values (normalized to the 0–1 scale) is shown in Figure 5. The mean DSC across all 61 studies was 0.893 (SD = 0.072), with a median of 0.908 and a range of 0.657 to 0.999. The distribution was left-skewed: 80% of studies reported DSC > 0.85, and 51% exceeded 0.90.

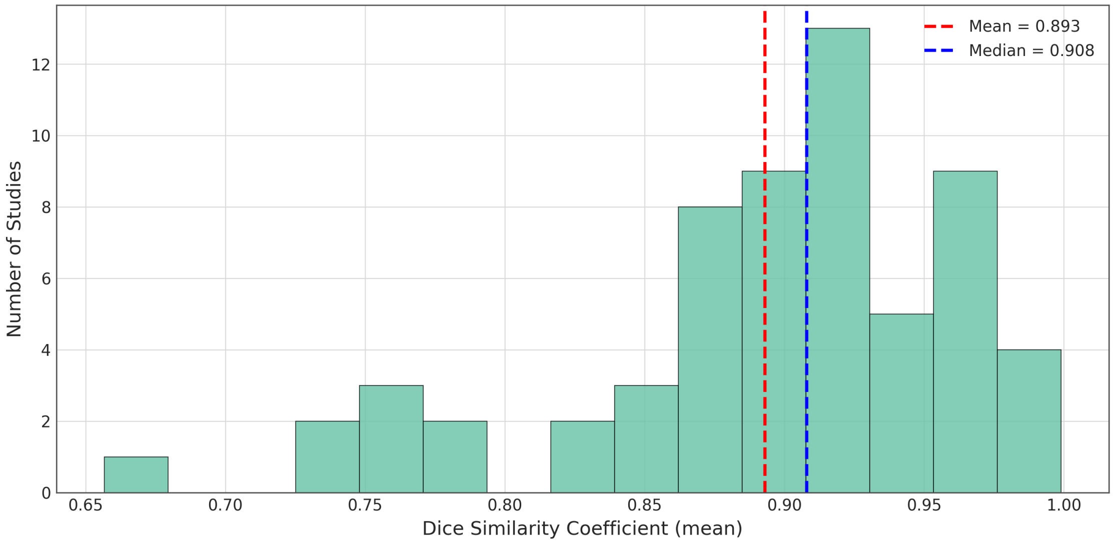

Figure 5. Distribution of Dice Similarity Coefficient (DSC) values across 61 studies reporting this metric. The distribution is left-skewed with a mean of 0.893 (red dashed line) and median of 0.908 (blue dashed line).

Forty-three studies (20.2%) reported the area under the receiver operating characteristic curve (AUC), with a mean of 0.960 across classification and detection tasks. AUC was reported almost exclusively by classification-focused studies using Kaggle-sourced datasets.

3.5.2 Heterogeneity Assessment

Formal heterogeneity analysis was restricted to the 15 studies reporting DSC mean, standard deviation, and sample size (Table 5). A DerSimonian–Laird random-effects model yielded a pooled mean DSC of 0.871 (95% CI: 0.852–0.889). Cochran's Q was 15,351.4 (df = 14, $p < 0.001$), and I^2 = 99.9%, indicating extreme heterogeneity (20). We note that Q is known to be overpowered in the presence of large-sample studies, which amplify even small true differences. The between-study standard deviation ($\hat{\tau}$ = 0.037) offers a more interpretable measure of dispersion: it implies that the true DSC in any given study could plausibly differ from the pooled mean by roughly ±0.04 on the 0–1 scale. The 95% prediction interval, representing the range within which the true effect of a future comparable study would be expected to fall, was 0.788 to 0.953, a much wider band than the confidence interval of the pooled mean. Given this degree of heterogeneity, the pooled mean is best read as a descriptive summary of reported performance in the published literature rather than as an estimate of a single underlying effect; the prediction interval is the more informative quantity for anticipating the performance of a future study.

Table 5. Random-effects heterogeneity assessment of DSC across 15 studies with paired mean, SD, and sample size.

Parameter	Value
Number of studies (k)	15
Pooled mean DSC (random-effects)	0.871
95% CI of pooled mean	[0.852, 0.889]
95% Prediction interval	[0.788, 0.953]
$\hat{\tau}^2$ (between-study variance)	0.00137
$\hat{\tau}$ (between-study SD)	0.037
Cochran's Q	15,351.4
Degrees of freedom	14
p-value (χ^2)	< 0.001
I^2	99.9%
Interpretation	Extreme heterogeneity

3.5.2.1 Sensitivity analysis.

Leave-one-out analysis, in which each study was sequentially removed and the pooled mean recomputed, produced estimates ranging from 0.863 (excluding Raza_2026) to 0.880 (excluding Jung_2026). No single study shifted the pooled estimate by more than 0.009, suggesting reasonable stability. Forest plot visualization of individual study effects with 95% SE-based confidence intervals is presented in Figure 6.

3.5.2.2 Missing-data sensitivity.

The complete-case pooled mean (0.871, k = 15) was slightly lower than the unweighted descriptive mean of all 61 DSC-reporting studies (0.893). Studies excluded from pooling for incomplete variance reporting therefore tended to report somewhat higher DSC values, indicating that the complete-case restriction is unlikely to have inflated the pooled estimate; if anything, it is conservative.

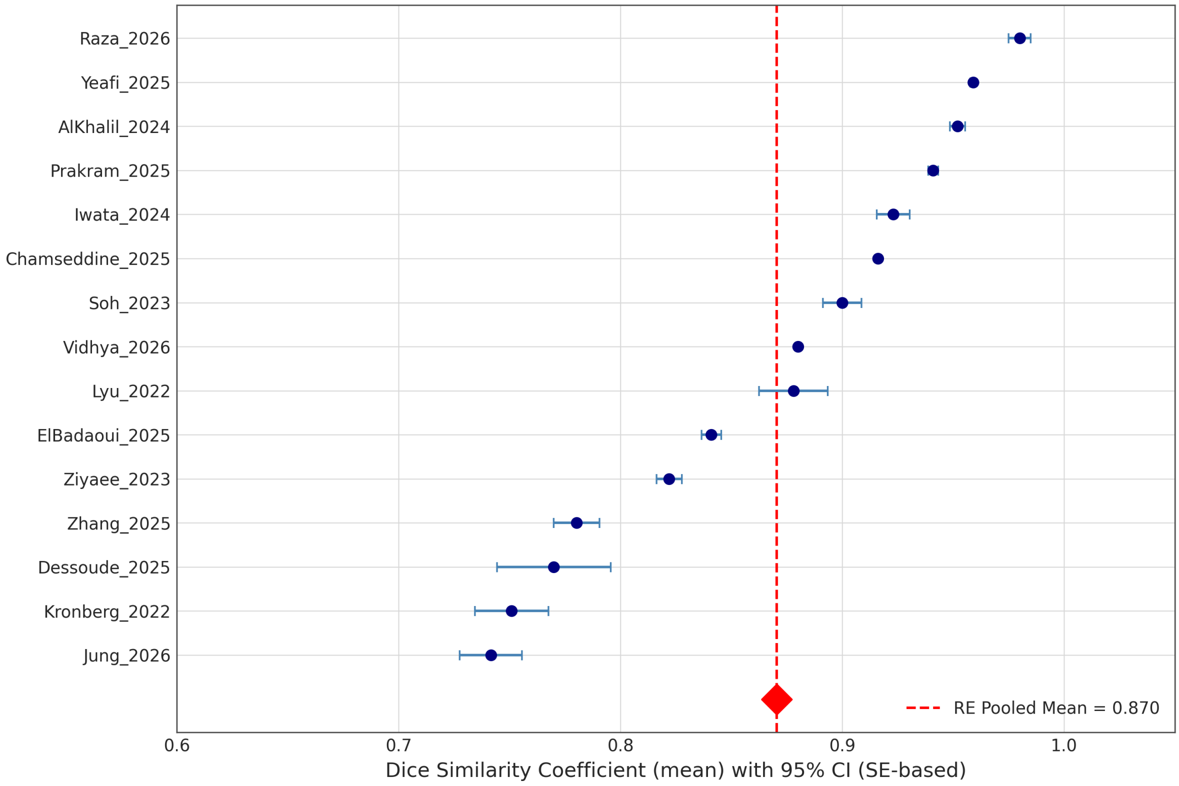

Figure 6. Forest plot showing individual study DSC values with 95% SE-based confidence intervals (mean $\pm$ 1.96 $\times$ SE, where SE = SD/$\sqrt{n}$) for the 15 studies reporting mean, standard deviation, and sample size. The red diamond indicates the random-effects pooled mean DSC (0.870 on the raw scale as plotted; the logit-based primary estimate is 0.871).

3.5.3 Publication Bias

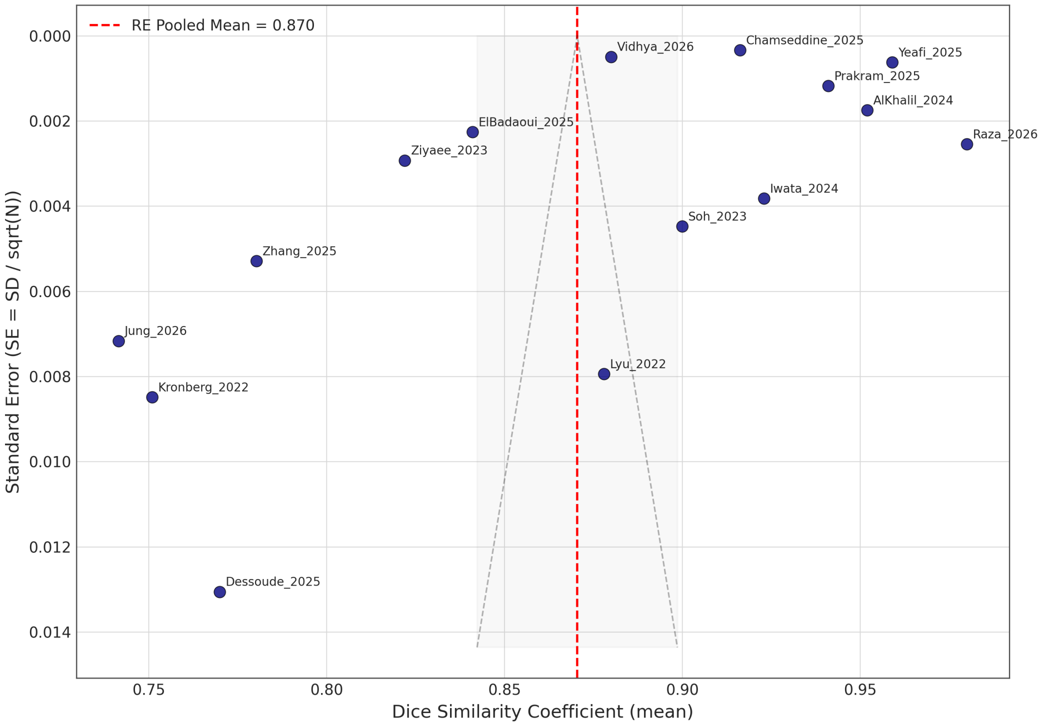

Figure 7. Funnel plot for publication bias assessment (N = 15 studies). The x-axis shows DSC and the y-axis shows standard error (inverted). The dashed red line indicates the random-effects pooled mean DSC (0.870 on the raw scale as plotted). Egger's regression test indicated statistically significant asymmetry (p = 0.003); see text for interpretation.

The funnel plot (Figure 7) was visibly asymmetric. High-precision studies (low SE) clustered at higher DSC values, while lower-precision studies were more dispersed and skewed toward lower values. Egger's regression test indicated statistically significant asymmetry (p = 0.003). Such asymmetry is consistent with small-study effects and reporting bias favoring positive results, but it is not specific to them: between-study heterogeneity of the magnitude observed here (I^2 = 99.9%) can itself produce funnel plot asymmetry when true effects differ systematically with study size. With only k = 15 studies, publication bias can therefore be neither confirmed nor excluded on this evidence. Studies with larger sample sizes and tighter confidence intervals tended to report higher DSC, while smaller or more variable studies reported a broader range of performance.

3.5.4 Subgroup Analyses

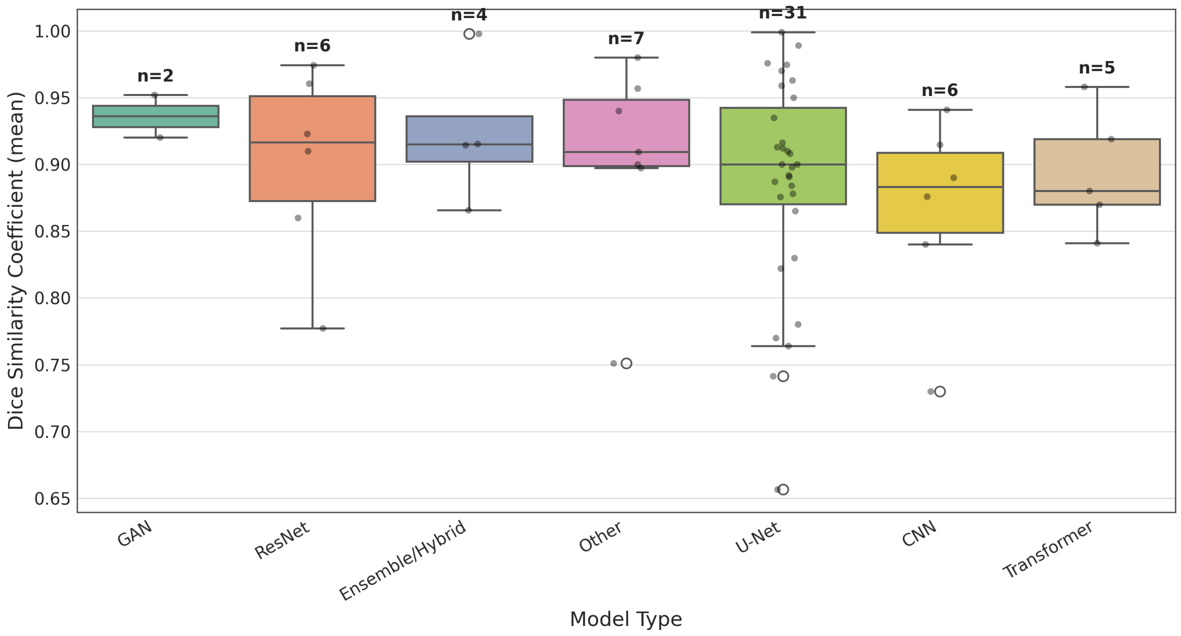

Figure 8. Box-and-whisker plot of DSC stratified by model architecture category, with individual data points overlaid. No statistically significant differences were observed across categories (Kruskal–Wallis H = 3.79, p = 0.705). Sample sizes are indicated above each box.

3.5.4.1 By model type.

The following subgroup analyses are exploratory and hypothesis-generating given the small and unbalanced group sizes. DSC did not differ significantly across architecture categories (Kruskal–Wallis H = 3.79, p = 0.705; Figure 8). Descriptive means and medians per category are reported in Table 6; with group sizes ranging from 2 to 31, the ordering of category means carries no evidential weight regarding architectural superiority.

Table 6. DSC subgroup analysis by model architecture category.

**Model Category**	N	**Mean DSC**	**Median DSC**
GAN	2	0.936	0.936
Ensemble / Hybrid	4	0.923	0.915
Other	7	0.905	0.909
ResNet	6	0.901	0.917
Transformer	5	0.894	0.880
U-Net	31	0.887	0.900
CNN	6	0.865	0.883
Kruskal–Wallis H = 3.79, p = 0.705 (not significant)			

3.5.4.2 By explainability.

The six studies with XAI had a slightly higher mean DSC (0.916) than the 55 without (0.891), but the difference was not significant (Mann–Whitney U = 193.5, p = 0.498).

3.5.4.3 By external validation.

Externally validated studies (n = 20) averaged a DSC of 0.905 versus 0.887 for internally validated studies (n = 41; Mann–Whitney U = 469.0, p = 0.369); the difference was not significant.

3.5.4.4 By task type.

DSC did not differ significantly across task categories (Kruskal–Wallis H = 2.50, p = 0.475). Combined segmentation-classification pipelines showed the highest mean DSC (0.915, n = 13), while pure segmentation studies reported a mean of 0.883 (n = 42). Classification tasks reported the highest mean AUC (0.960, n = 30).

3.5.5 Regression Analysis

Multiple regression analysis was performed on 45 studies with complete data for all predictor variables (Table 7). The overall model was not statistically significant (F = 0.563, p = 0.691), with R^2 = 0.053 (adjusted $R^2 = -0.041$): year, sample size, number of modalities, and explainability together explained only 5.3% of the variance in DSC. Consistent with the non-significant overall model, none of the individual coefficients reached statistical significance either (Table 7).

Table 7. OLS multiple regression results: predictors of DSC performance.

**Variable**	β	**SE**	t	p	**95% CI**
Intercept	−21.888	19.125	−1.144	0.259	[−60.54, 16.77]
Year	+0.011	0.009	1.190	0.241	[−0.008, 0.030]
Sample size	−0.000	0.000	−0.455	0.652	[−0.000, 0.000]
Modalities (N)	+0.004	0.009	0.466	0.644	[−0.013, 0.021]
Explainability	+0.030	0.036	0.836	0.408	[−0.043, 0.104]
R^2 = 0.053; Adjusted $R^2 = -0.041$; F = 0.563, p = 0.691					

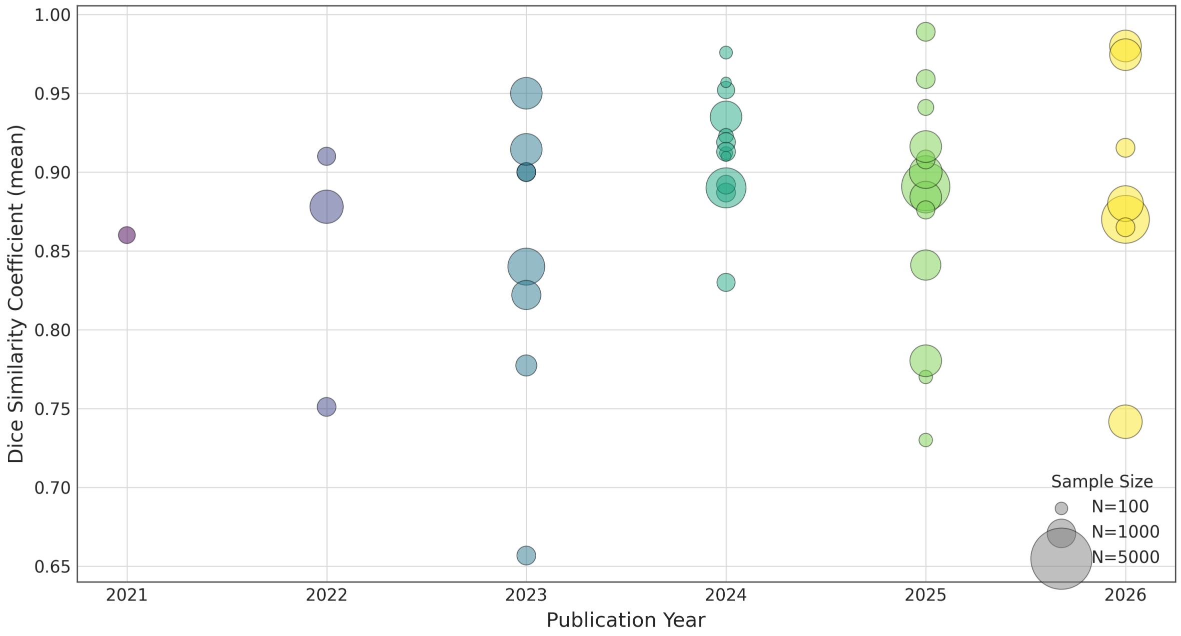

Figure 9. Bubble plot showing the relationship between publication year (x-axis), DSC (y-axis), and total sample size (bubble diameter).

The bubble plot (Figure 9) shows large within-year variance in DSC and no clear upward trend over time.

3.6 In-Depth Analysis of Selected Studies

Beyond aggregate statistics, we examined eight studies in greater depth (Table 8). We chose these for their methodological rigor, architectural originality, or clinical relevance, specifically the presence of external validation, explainability components, multi-institutional data, or otherwise distinctive contributions.

Two studies stood out for combining high segmentation accuracy with explainability. Khushubu et al. (23) proposed TransUNetB, a hybrid Transformer–U-Net that reached a DSC of 0.989 on BraTS 2020/2021 while providing built-in interpretability. Chamseddine et al. (24) took a different approach with a Gabor-enhanced 2D U-Net using squeeze-and-excitation blocks and attention gates (DSC = 0.916, SD = 0.012), validated across four BraTS editions (2018–2021).

On the federated learning front, Raza et al. (25) showed that weight sparsification and clustering on the FeTS 2024 dataset can yield a DSC of 0.980 without centralizing patient data. The classification side was represented by Garcia-Ruiz et al. (26), whose DISCERN system distinguished GBM, metastasis, and PCNSL from perfusion MRI across multiple centers (AUC = 0.83), and by Afroj et al. (27), whose MobDenseNet (MobileNetV1–DenseNet121 hybrid) achieved an AUC of 0.999 on cross-dataset evaluation.

Not all results were positive. Saeed et al. (28) found that none of the tested architectures (ResNets, DenseNets, EfficientNets, Swin Transformers) could reliably predict MGMT promoter methylation from MRI alone, an important negative finding for the radiogenomics community. Among synthesis-oriented work, Al-Fakih et al. (29) used SE-GAN for FLAIR synthesis paired with nnU-Net segmentation, externally validated on BraTS Africa 2023; segmentation with synthesized FLAIR reached a DSC of 0.873, versus 0.901 with real FLAIR and 0.688 with FLAIR missing. Finally, Ganie et al. (30) built an explainable

hybrid framework around stacked transfer learning and a PD-CNN, integrating three complementary XAI methods (SHAP, Grad-CAM, and Score-CAM) to achieve an AUC of 0.989.

Table 8. Detailed characteristics of eight selected studies of particular methodological interest. DSC = Dice Similarity Coefficient; ExtVal = External Validation; XAI = Explainable AI. A dash (—) indicates a metric not applicable to the study's task (e.g., DSC for classification studies); NR = not reported in the original publication. N = 1251 for Chamseddine et al., Raza et al., and Al-Fakih et al. refers in each case to the BraTS 2021 training cohort (1251 subjects), on which all three studies are based: FeTS 2024 repackages this cohort for federated partitioning, and the cross-edition and BraTS Africa evaluations are additional test sets drawn beyond it.

Study	Architecture	Task	Dataset	N	DSC	AUC	ExtVal	XAI
Khushubu et al. (23)	TransUNetB	Segmentation	BraTS 2020/2021	369	0.989	—	Yes	Yes
Chamseddine et al. (24)	Gabor U-Net + SE + AG	Segmentation	BraTS 2018–2021	1251	0.916	—	Yes	Yes
Raza et al. (25)	FedWSOComp	Federated Seg.	FeTS 2024 (test n = 67)	1251	0.980	—	Yes	No
Garcia-Ruiz et al. (26)	DISCERN (1D CNN)	Classification	Multi-center	568	—	0.83	Yes	Yes
Saeed et al. (28)	ResNet/Swin/ViT	Radiogenomics	RSNA-MICCAI/TCIA	585	—	—	No	Yes
Al-Fakih et al. (29)	SE-GAN + nnU-Net	Synthesis + Seg.	BraTS + Africa	1251	0.873	—	Yes	No
Afroj et al. (27)	MobDenseNet	Classification	Figshare/Sartaj	3064	—	0.999	Yes	No
Ganie et al. (30)	Stacked TL + PD-CNN	Classification	NR	NR	—	0.989	No	Yes

3.7 Methodological Quality Assessment

3.7.1 Explainability

Only 40 of 213 studies (18.8%) incorporated any explainable AI method. The most common XAI techniques were Grad-CAM (31, 32, 30), SHAP (33, 32), and LIME (34, 35). A few studies combined multiple XAI methods: Nazir et al. (31) combined SHAP, LIME, and Grad-CAM; Ganie et al. (30) employed SHAP, Grad-CAM, and Score-CAM; and Alim et al. (32) used Grad-CAM with SHAP. The vast majority of studies (130, 61.0%) explicitly reported no explainability component, and 43 studies (20.2%) did not address explainability at all. Table 9 summarizes the XAI methods used across the identified studies.

Table 9. Studies incorporating explainable AI (XAI) methods, as identified from the systematic review. Multiple XAI techniques are listed where applicable.

Study	XAI Method(s)	Model Type
Nazir et al. (31)	SHAP + LIME + Grad-CAM	Custom CNN
Ganie et al. (30)	SHAP + Grad-CAM + Score-CAM	Stacked TL + PD-CNN
Alim et al. (32)	Grad-CAM + SHAP	VGG16/Xception knowledge distillation
Agrawal and Chaki (34)	LIME	CerebralNet (MobileNetV2 + ASPP)
Narasimha Raju et al. (35)	LIME	Ensemble CNNs + GLCM + XGBoost
Bhuiyan et al. (33)	SHAP	ResNet50 + PCA + XGBoost
Ayaz et al. (36)	Volumetric attention maps	3D CNN + PCA
Khushubu et al. (23)	Yes (unspecified)	TransUNetB
Gagliardi et al. (37)	Yes (unspecified)	VGG16 + hand-crafted features + KNN
Saeed et al. (28)	Yes (unspecified)	ResNets/DenseNets/EfficientNets/Swin/ViT
Saeed et al. (38)	Yes (unspecified)	ResNets/DenseNets/EfficientNets/Swin/ViT
Dang et al. (39)	Yes (unspecified)	UNet + VGG/GoogleNet
Hasan et al. (40)	Yes (unspecified)	Ensemble CNN
Ahmed et al. (41)	Yes (unspecified)	SwinResDual (Swin + ResNet50)
Appiah et al. (42)	Yes (unspecified)	POD-CNN

3.7.2 External Validation

External validation (testing the trained model on an independent dataset not used during training or development) was performed in only 40 of 213 studies (18.8%). The remaining 166 studies (77.9%) relied exclusively on internal validation (hold-out splits or cross-validation on the same dataset), and 7 studies did not report their validation approach. “External validation” encompasses a spectrum of evidence levels: (1) internal hold-out or cross-validation within the same dataset, (2) cross-dataset validation using different public benchmarks (e.g., training on BraTS 2020 and testing on BraTS 2021), and (3) multi-institutional clinical validation on prospectively or retrospectively collected data from independent sites. Most of the 40 externally validated studies fell into level (2); true multi-institutional clinical validation (level 3) was rare. Table 10 presents the subset of externally validated studies identified as methodologically exemplary, highlighting their validation datasets, sample sizes, and primary metrics.

Table 10. Selected studies with external validation, showing validation dataset and primary performance metric. DSC and AUC are reported where available. A dash (—) indicates a metric not applicable to the study's task; NR = not reported in the original publication. N refers to the development cohort size as extracted, not to the external test set, which is typically smaller (e.g., the independent FeTS 2024 test set comprises 67 patients).

Study	External Validation Dataset	N	DSC	AUC
Raza et al. (25)	FeTS 2024 (multi-institutional federated)	1251	0.980	—
Chamseddine et al. (24)	BraTS 2018–2021 (cross-edition)	1251	0.916	—
Al Khalil et al. (43)	BraTS 2020 (Fourier domain adaptation)	369	0.952	—
Yeafi et al. (44)	BraTS 2020 (survival prediction)	369	0.959	—
Rastogi et al. (45)	Multi-center undersampled MRI	802	0.890	—
Al-Fakih et al. (29)	BraTS Africa 2023	1251	0.873	—
Garcia-Ruiz et al. (26)	Multi-center perfusion MRI	568	—	0.830
Afroj et al. (27)	Figshare + Sartaj (cross-dataset)	3064	—	0.999
Guan et al. (46)	Cross-institutional MRI	NR	—	NR
Zhou et al. (47)	Synthetic tumor generation	NR	—	—
Saeed et al. (28)	TCIA/TCGA-GBM/ACRIN-6684	585	—	—

3.7.3 Robustness Testing

No study reported extractable robustness metrics according to our predefined extraction fields for noise degradation, resolution degradation, or domain-shift performance drops. These columns were blank across all 213 studies. While some studies may have performed informal robustness checks not captured by our extraction template, no study systematically reported how performance changed under controlled input perturbations (noise injection, resolution variation) or when applied to data from different scanners, institutions, or acquisition protocols. The implications for clinical translation, where imaging conditions vary widely, are discussed below.

3.7.4 Data Quality and Reporting

Reporting quality was uneven. Scale inconsistency (0–1 vs. 0–100) affected both DSC and AUC values. Only 61 of 213 studies (28.6%) reported DSC at all, and only 16 (7.5%) reported both DSC mean and standard deviation; of these, 15 also reported sample size, which made formal random-effects pooling possible. This severely limited the scope of formal meta-analysis. Dataset naming conventions varied wildly, complicating cross-study comparisons, and 16 studies (7.5%) did not report which dataset they used.

4 DISCUSSION

4.1 Principal Findings

This systematic review, covering 213 studies published between January 2018 and March 2026, is the broadest synthesis of ML approaches we are aware of to MRI-based brain tumor analysis to date, encompassing segmentation, classification, detection, grading, radiogenomics, prognosis, and synthesis. The picture is one of rapid growth and high reported accuracy, but persistent methodological weaknesses stand between benchmark results and clinical use.

Several of the highest-performing DSC studies illustrate the features associated with strong segmentation results. Khushubu et al. (23) achieved a DSC of 0.989 with TransUNetB, a hybrid Transformer–U-Net with built-in interpretability, on BraTS 2020/2021. Raza et al. (25) reached 0.980 with federated weight sparsification on the multi-institutional FeTS 2024 dataset. Yeafi et al. (44) reported 0.959 on BraTS 2020, and Al Khalil et al. (43) achieved 0.952 using Fourier domain adaptation for multi-modal synthesis. Al-Fakih et al. (29) illustrate the value of external validation in this group: their SE-GAN-based FLAIR synthesis paired with nnU-Net, tested on BraTS Africa 2023, reached a DSC of 0.873 with synthesized FLAIR versus 0.901 with real FLAIR. These studies shared standardized BraTS-derived datasets, multi-modal MRI inputs, encoder–decoder or hybrid architectures, and extensive preprocessing. However, high DSC values should not be interpreted as direct evidence of clinical readiness, particularly when evaluation is limited to curated public datasets. By contrast, the lowest-performing studies (DSC values down to 0.657) tended to involve more heterogeneous or smaller institutional datasets, single-modality inputs, less standardized preprocessing, harder tumor subregions such as non-enhancing tumor core, or less mature validation protocols. This contrast suggests that performance differences reflect dataset and task difficulty and evaluation design as much as model architecture. The clustering of reported DSC values near 0.90 across the wider corpus likely reflects the maturity of BraTS-based segmentation benchmarks.

The meta-analytic results must be read with the extreme heterogeneity (I^2 = 99.9%) in the foreground. The pooled mean DSC of 0.871 is best treated as a descriptive summary of reported performance; the 95% prediction interval of 0.788 to 0.953 better conveys what a new study should expect. This dispersion has identifiable sources: different BraTS editions and institutional datasets, different tumor sub-region definitions (whole tumor versus enhancing tumor versus tumor core), two-dimensional versus three-dimensional evaluation, heterogeneous preprocessing and annotation protocols, and internal versus external test conditions. Averaging across these settings mixes genuinely different estimands, which is why the sources of variance deserve more attention than any single pooled number.

That U-Net variants still lead (19.2%) shows that the encoder-decoder design remains dominant with skip connections for medical image segmentation (7, 13). At the same time, the rapid uptake of Transformer-based architectures (9.4%) reflects growing interest in attention-based models (9, 10). Hybrid CNN-Transformer models, which combine local feature extraction with global self-attention, are a particularly active area, as illustrated by Vidhya et al. (48), Kaur et al. (49), and Nguyen-Tat et al. (50).

At a finer grain than these category labels, specific architectural mechanisms map onto specific segmentation difficulties. Attention gates, as used in the dual-attention Gabor-enhanced U-Net of Chamseddine et al. (24), suppress irrelevant background activations propagated through skip connections and thereby sharpen the boundaries of small structures such as the enhancing tumor core. Squeeze-and-excitation blocks reweight feature channels, which helps separate tissue classes with overlapping intensity profiles, such as edema and non-enhancing tumor. Multi-scale feature fusion, exemplified by the stacked framework of Ganie et al. (30), combines coarse contextual features with fine spatial detail and benefits the thin, irregular margins of the non-enhancing tumor core. Global self-attention in Transformer hybrids (23, 50) captures the long-range spatial context relevant to spatially extended peritumoral edema. That these mechanisms plausibly target the hardest sub-regions, while category-level DSC differences remain non-significant, reinforces a central conclusion of this review: gains from architectural components are contingent on data, preprocessing, and training discipline rather than guaranteed by the component itself.

The absence of significant DSC differences across architecture categories (Kruskal–Wallis p = 0.705) warrants closer examination. It implies that architecture choice matters less than implementation details: preprocessing, data augmentation, loss function selection, and hyperparameter optimization. nnU-Net made

a similar point (13): disciplined optimization of the training pipeline can matter more than the network topology itself. The regression analysis points the same way: year, sample size, modality count, and explainability jointly explained only 5.3% of the variance in DSC, indicating that performance hinges on study-specific factors (dataset selection, preprocessing, hyperparameter tuning, and evaluation protocol) rather than broad study-level variables.

4.2 Comparison with Prior Reviews

Table 1 situates this review among recent syntheses. The only directly comparable quantitative synthesis is that of Wang et al. (18), who pooled segmentation performance across 19 externally validated studies published between 2015 and 2023. Our corpus is an order of magnitude larger (213 studies), extends through March 2026, and is not restricted to externally validated work; our pooled DSC of 0.871 is consistent with their finding of high aggregate segmentation accuracy, while our prediction interval (0.788–0.953) quantifies a dispersion that a confidence interval alone conceals. Descriptive reviews (15, 16, 17, 14) have documented the same architectural trends we observe, namely the dominance of U-Net variants and the rise of Transformer hybrids, but could not quantify performance or its heterogeneity. Beyond corpus size and pooling, the present review adds three elements absent from all prior syntheses in Table 1: a structured audit of explainability and external validation prevalence across all task types, a systematic attempt to extract robustness metrics (which returned none, itself an informative finding), and an explicit separation of segmentation and classification endpoints.

4.3 Methodological Gaps: Explainability, External Validation, and Robustness

4.3.0.1 Explainability.

That only 18.8% of studies incorporated any form of explainability limits clinical adoption. Clinicians need to understand why a model reached a given conclusion before they will trust it, and regulators now expect such interpretability. Among the few studies that did address explainability, most relied on post-hoc visualization (Grad-CAM), which provides only surface-level insight. Deeper approaches such as concept-based explanations, uncertainty quantification, and inherently interpretable architectures remain largely unexplored.

The studies that have invested in XAI (23, 30, 28, 38, 40, 37) show that adding explainability does not necessarily degrade performance. Our subgroup analysis, in fact, showed a non-significant trend toward higher DSC in XAI-positive studies (0.916 vs. 0.891, $p = 0.498$), hinting that groups who invest in explainability also tend to produce stronger work overall.

4.3.0.2 External validation.

Only 18.8% of studies performed external validation, which calls generalizability into question. Internal validation on a single dataset, even with cross-validation, cannot capture the distributional shifts that arise in multi-institutional practice: different scanners, acquisition protocols, patient populations, and tumor characteristics. This deficit is compounded by the complete absence of robustness testing against noise and domain shift. Selection effects may also color the externally validated subset: the non-significantly higher mean DSC among externally validated studies (0.905 versus 0.887) may partly reflect that groups with stronger-performing methods are more inclined to seek external validation.

Among the studies that did validate externally (25, 26, 24, 45, 29), performance on held-out data was typically lower than on internal benchmarks, exactly the kind of drop that internal-only evaluation conceals.

Federated learning (25, 51, 52) offers one way forward, allowing multi-institutional model development without centralizing patient data.

External validation should be reported with greater granularity. Cross-validation within the same dataset, testing on a held-out split, cross-edition evaluation within the BraTS ecosystem, cross-public-dataset validation, and multi-institutional clinical validation represent different levels of evidence. Future studies should explicitly label the validation level and report performance degradation relative to internal validation, because such degradation is directly relevant to clinical generalizability.

4.3.0.3 Robustness.

Robustness should be elevated from an optional experiment to a core evaluation requirement. MRI-based tumor models are exposed to scanner differences, acquisition protocol variation, motion artifacts, intensity non-standardization, missing sequences, and domain shift across institutions. Therefore, clinically oriented studies should report performance under controlled perturbations such as noise injection, resolution changes, missing modalities, intensity shifts, and external-domain testing. Without such reporting, high benchmark performance remains insufficient evidence for deployment readiness. The complete absence of extractable robustness metrics across all 213 studies in our sample represents a serious gap that the field must address.

4.4 Dataset Ecosystem and Publication Bias

There is a trade-off between benchmark standardization and clinical relevance in dataset selection. BraTS challenge datasets offer standardized preprocessing, consistent annotations, and community-wide comparability, but they cover a narrow slice of clinical reality (predominantly gliomas from tertiary centers). Kaggle-hosted datasets are accessible and popular for classification, yet they often lack provenance documentation, annotation quality assurance, and the clinical context expected of rigorous medical imaging research.

The extreme fragmentation of dataset naming conventions alone complicates meta-analysis and points to the need for standardized identification schemes. Future studies should adopt persistent dataset identifiers (DOIs) and describe acquisition parameters, annotation protocols, and inclusion criteria in full.

The funnel plot asymmetry we observed is consistent with publication bias favoring positive results, the well-documented “file drawer” effect in which negative or null findings are less likely to reach print. It is, however, not conclusive evidence of such bias: as noted in Section 3, between-study heterogeneity of the magnitude observed here can itself generate funnel plot asymmetry, and with $k = 15$ studies the two explanations cannot be separated. Whichever mechanism dominates, the practical implication is the same: combined with the general absence of external validation and robustness testing, the published literature likely paints a more favorable picture of these methods than clinical practice would.

4.5 Clinical Implications: False Negatives, False Positives, and the Limits of Aggregate Metrics

A clinically meaningful interpretation of model performance requires distinguishing false-negative and false-positive errors. In classification and detection studies, sensitivity and specificity reflect different clinical priorities. False negatives may delay diagnosis, referral, or treatment planning, whereas false positives may increase unnecessary follow-up imaging, radiology workload, patient anxiety, and downstream confirmatory procedures. The optimal operating point is therefore context-dependent: triage applications may tolerate more false positives to minimize missed tumors, while treatment-planning applications require higher specificity and spatial precision. AUC alone, without a specified operating

threshold, is insufficient for clinical decision-making; future studies should report sensitivity, specificity, positive and negative predictive values, and calibration at clinically relevant thresholds.

For segmentation, the analogous distinction is between under-segmentation and over-segmentation. False-negative tumor voxels can underestimate enhancing tumor, tumor core, or peritumoral edema, potentially affecting volumetric assessment, surgical planning, radiation target definition, and response monitoring. False-positive voxels can overestimate tumor extent and reduce the clinical usefulness of automated contours. Because a high DSC may still conceal clinically relevant boundary errors, future studies should complement DSC with HD95, surface Dice, lesion-wise sensitivity, subregion-specific metrics, uncertainty estimates, and reader studies that evaluate how automated outputs change clinical decisions.

4.6 Limitations

This study has several limitations:

1. The review protocol was retrospectively registered on OSF (DOI: 10.17605/OSF.IO/H4YF7) after the search had commenced, rather than prospectively registered in PROSPERO.
2. Risk of bias was assessed using two complementary instruments: a custom 8-item reporting checklist for all 213 studies and formal PROBAST+AI scoring for the 15 meta-analysis studies. While this provides broader coverage than a single tool, the custom checklist lacks the formal validation of QUADAS-2 or PROBAST.
3. The meta-analysis was constrained by the small number of studies reporting paired DSC mean and standard deviation (15 of 213), limiting statistical power. Although a logit transformation was applied to address the bounded nature of DSC, the small k means that heterogeneity estimates and confidence intervals should be interpreted with caution.
4. The OLS regression used untransformed DSC values despite DSC being a bounded metric; however, given the descriptive intent and the non-significant results, this is unlikely to affect conclusions.
5. The high-level categorization of model types sacrifices detail about specific architectural innovations.
6. The restriction to English-language publications may have introduced language bias.
7. The rapid pace of publication in this field means that studies published after our search cutoff (16 March 2026) are not included.
8. The complete absence of extractable robustness data across all 213 studies meant that this dimension could not be analyzed quantitatively.
9. A formal GRADE certainty-of-evidence assessment was not performed, as meaningful application would require separate evidence bodies for each task type with task-specific outcomes and risk-of-bias considerations.

4.7 Recommendations for Future Research

We make seven recommendations:

1. **Mandatory external validation.** Every study should test its model on at least one independent dataset not seen during development, to show generalizability beyond the training distribution.
2. **Robustness testing.** No study in our sample reported performance under noise injection, resolution variation, or domain shift. Robustness evaluation under controlled perturbations should become a minimum requirement for any clinically oriented study.
3. **Explainability from the start.** XAI methods should be built into model development and evaluation rather than bolted on after the fact.

4. **Standardized reporting.** At a minimum, studies should report DSC with standard deviation, specify datasets using persistent identifiers, and follow PRISMA-compliant guidelines to support reproducibility.
5. High DSC or AUC on a benchmark does not prove **clinical validation**. The field needs reader studies comparing human and automated segmentation, time-savings analyses, and workflow integration experiments that measure whether these tools help in practice.
6. **Multi-institutional collaboration.** Federated learning and data-sharing initiatives must expand if the field is to close the generalization gap.
7. Relative to segmentation and classification, tasks such as radiogenomics, survival prediction, and treatment response monitoring remain **under-explored**, despite their potential clinical value.

5 CONCLUSIONS

This review synthesized 213 studies of machine learning for MRI-based brain tumor analysis published between January 2018 and March 2026. Reported performance is high across tasks: segmentation studies cluster near a DSC of 0.9, and classification studies report a descriptive mean AUC of 0.960. The random-effects synthesis, however, shows how wide the plausible range remains. Although the pooled mean DSC was 0.871, the 95% prediction interval of 0.788 to 0.953 spans the difference between clinically useful and clinically unusable segmentation, and heterogeneity was extreme. No architecture category significantly outperformed any other, indicating that training data, preprocessing, and evaluation design shape reported performance more than the choice of network.

For clinical practice, these findings carry a direct message. An automated contour whose true performance may lie anywhere within the prediction interval cannot yet be trusted for neurosurgical planning or radiotherapy target definition without site-specific validation. The deficits documented here explain why: 81.2% of studies provide no explainability, 77.9% never test outside their development dataset, and none report robustness under scanner, protocol, or population shift, which are precisely the conditions a deployed tool would face. Until these gaps close, high benchmark scores should be read as evidence of feasibility rather than of readiness, and adoption decisions should demand site-relevant external validation, calibration at clinically meaningful operating points, and interpretable outputs that radiologists and surgeons can verify.

The path forward is actionable. Standardized reporting of variance and sample size would allow future syntheses to pool far more than 15 of 61 DSC-reporting studies. Multi-institutional collaboration, including federated learning, can supply the diverse data that generalization requires. Robustness testing and explainability must move from optional extras to core evaluation requirements, and under-explored tasks such as radiogenomics and treatment response monitoring deserve attention commensurate with their clinical value. The architectures are ready; the evidence standards are not. Closing that gap is now the field's central task.

CONFLICT OF INTEREST STATEMENT

The authors declare that the research was conducted in the absence of any commercial or financial relationships that could be construed as a potential conflict of interest.

AUTHOR CONTRIBUTIONS

Conceptualization, A.M.K. and I.V.B.; Methodology, A.M.K. and M.D.K.; Software, M.D.K.; Validation, A.M.K., Z.B. and D.A.B.; Formal Analysis, M.D.K. and A.M.K.; Investigation, A.M.K., Z.B., D.A.B., M.Z. and M.D.K.; Resources, A.M.K.; Data Curation, M.D.K. and A.M.K.; Writing Original Draft Preparation, A.M.K. and M.D.K.; Writing Review & Editing, A.M.K., V.V.B., D.O.M. and I.V.B.; Visualization, M.D.K.; Supervision, A.M.K.; Project Administration, A.M.K. All authors have read and agreed to the published version of the manuscript.

FUNDING

This research was funded by the Committee of Science of the Ministry of Science and Higher Education of the Republic of Kazakhstan (Grant No. AP23486255).

ETHICS STATEMENT

Institutional review board statement: Not applicable. Informed consent statement: Not applicable.

ACKNOWLEDGMENTS

The authors acknowledge the use of Python 3.12 with pandas, NumPy, SciPy, statsmodels, matplotlib, and seaborn libraries for statistical analysis and visualization.

SUPPLEMENTAL DATA

The following supporting information is available: File S1: PRISMA 2020 Checklist; File S2: Verbatim search strings for all databases; File S3: Full data extraction table (213 studies $\times$ 88 variables, Excel/CSV); File S4: PROBAST+AI risk-of-bias assessment for the 15 meta-analysis studies; File S5: Sensitivity analyses (REML, Hartung–Knapp, raw-scale pooling).

DATA AVAILABILITY STATEMENT

The dataset generated and analyzed for this systematic review, including the full extraction table of 213 studies across 88 variables, is available from the corresponding author upon reasonable request. All included primary studies are cited in the reference list with their respective DOIs.

ABBREVIATIONS

The following abbreviations are used in this manuscript:

ML	Machine Learning
MRI	Magnetic Resonance Imaging
DSC	Dice Similarity Coefficient
CNN	Convolutional Neural Network
XAI	Explainable Artificial Intelligence
AUC	Area Under the Curve
GBM	Glioblastoma
PRISMA	Preferred Reporting Items for Systematic Reviews and Meta-Analyses
BraTS	Brain Tumor Segmentation
FLAIR	Fluid-Attenuated Inversion Recovery
GAN	Generative Adversarial Network
ViT	Vision Transformer
HD95	95th percentile Hausdorff Distance
OLS	Ordinary Least Squares
SVM	Support Vector Machine
TCIA	The Cancer Imaging Archive

REFERENCES

1.Louis D, Perry A, Wesseling P, Brat D, Cree I, Ellison D. The 2021 WHO classification of tumors of the central nervous system: a summary. *Neuro-Oncology* **23** (2021) 1231–1251. doi:10.1093/neuonc/noab106.

2.Bray F, Laversanne M, Sung H, Ferlay J, Siegel R, Soerjomataram I, et al. Global cancer statistics 2022: GLOBOCAN estimates of incidence and mortality worldwide for 36 cancers in 185 countries. *CA: A Cancer Journal for Clinicians* **74** (2024) 229–263. doi:10.3322/caac.21834.

3.Ostrom Q, Price M, Neff C, Cioffi G, Waite K, Kruchko C, et al. CBTRUS Statistical Report: Primary Brain and Other CNS Tumors Diagnosed in the United States in 2017–2021. *Neuro-Oncology* **26** (2024) vi1–vi85. doi:10.1093/neuonc/noae145.

4.Stupp R, Mason W, van den Bent M, Weller M, Fisher B, Taphoorn M, et al. Radiotherapy plus concomitant and adjuvant temozolomide for glioblastoma. *New England Journal of Medicine* **352** (2005) 987–996. doi:10.1056/NEJMoa043330.

5.Menze B, Jakab A, Bauer S, Kalpathy-Cramer J, Farahani K, Bakas S. The Multimodal Brain Tumor Image Segmentation Benchmark (BRATS). *IEEE Transactions on Medical Imaging* **34** (2015) 1993–2024. doi:10.1109/TMI.2014.2377694.

6.LeCun Y, Bengio Y, Hinton G. Deep learning. *Nature* **521** (2015) 436–444. doi:10.1038/nature14539.

7.Ronneberger O, Fischer P, Brox T. U-Net: Convolutional networks for biomedical image segmentation. *MICCAI 2015* (2015) 234–241. doi:10.1007/978-3-319-24574-4_28.

8.He K, Zhang X, Ren S, Sun J. Deep residual learning for image recognition. *CVPR* (2016) 770–778. doi:10.1109/CVPR.2016.90.

9.Dosovitskiy A, Beyer L, Kolesnikov A, Weissenborn D, Zhai X, Unterthiner T, et al. An image is worth 16x16 words: Transformers for image recognition at scale. *ICLR* (2021).

10.Vaswani A, Shazeer N, Parmar N, Uszkoreit J, Jones L, Gomez A, et al. Attention is all you need. *NeurIPS* **30** (2017) 5998–6008.

11.Baid U, Ghodasara S, Mohan S, Bilello M, Calabrese E, Colak E, et al. The RSNA-ASNR-MICCAI BraTS 2021 benchmark on brain tumor segmentation and radiogenomics. *arXiv preprint arXiv:2107.02314* (2021).

12. Dice L. Measures of the amount of ecologic association between species. *Ecology* **26** (1945) 297–302. doi:10.2307/1932409.

13. Isensee F, Jaeger P, Kohl S, Petersen J, Maier-Hein K. nnU-Net: a self-configuring method for deep learning-based biomedical image segmentation. *Nature Methods* **18** (2021) 203–211. doi:10.1038/s41592-020-01008-z.

14. Pasunoori K, Prasad C, Raj Kumar K. A systematic review on deep learning based brain tumor segmentation and detection using MRI: Past insights, present techniques and future trends. *Computational Biology and Chemistry* **120** (2026) 108696. doi:10.1016/j.compbiolchem.2025.108696.

15. Satushe V, Vyas V, Metkar S, Singh D. AI in MRI brain tumor diagnosis: A systematic review of machine learning and deep learning advances (2010–2025). *Computers in Biology and Medicine* (2025). doi:10.1016/j.compbiomed.2025.109991.

16. Fahim T, Alam F, Hossain M. Brain tumor detection, classification and segmentation by deep learning models from MRI images: Recent approaches, challenges and future directions. *Array* **28** (2025) 100571. doi:10.1016/j.array.2025.100571.

17. Dorfner F, et al. A review of deep learning for brain tumor analysis in MRI. *npj Precision Oncology* (2025).

18. Wang T, Shiao Y, Hong J, Lee W, Chen M, Hsu H, et al. Artificial Intelligence Detection and Segmentation Models: A Systematic Review and Meta-Analysis of Brain Tumors in Magnetic Resonance Imaging. *Mayo Clinic Proceedings: Digital Health* (2024). doi:10.1016/j.mcpdig.2024.01.002.

19. Page M, McKenzie J, Bossuyt P, Boutron I, Hoffmann T, Mulrow C, et al. The PRISMA 2020 statement: an updated guideline for reporting systematic reviews. *BMJ* **372** (2021) n71. doi:10.1136/bmj.n71.

20. Higgins J, Thompson S, Deeks J, Altman D. Measuring inconsistency in meta-analyses. *BMJ* **327** (2003) 557–560. doi:10.1136/bmj.327.7414.557.

21. Tejani A, Klontzas M, Gatti A, et al. Checklist for Artificial Intelligence in Medical Imaging (CLAIM): 2024 Update. *Radiology: Artificial Intelligence* **6** (2024) e240300.

22. Moons K, et al. PROBAST+AI: Updated quality, risk of bias, and applicability assessment for prediction models and algorithms. *BMJ* (2025).

23. Khushubu KG, Masum AA, Rahman MH, Hasan SMS, Bhuiyan MIH, Mahmud MR, et al. TransUNetB: An advanced Transformer-UNet framework for efficient and explainable brain tumor segmentation. *Informatics in Medicine Unlocked* (2025). doi:10.1016/j.imu.2025.101706.

24. Chamseddine E, Tlig L, Chaari L, Sayadi M. A Gabor-enhanced deep learning approach with dual-attention for 3D MRI brain tumor segmentation. *Computers in Biology and Medicine* (2025). doi:10.1016/j.compbiomed.2025.111047.

25. Raza A, Raggio C, Guzzo A, Spadea M, Fortino G. Towards robust neurocomputing model in efficient federated brain tumour segmentation with sparsification and weights clustering. *Neurocomputing* (2026). doi:10.1016/j.neucom.2026.133142.

26. Garcia-Ruiz A, Pons-Escoda A, Grussu F, Naval-Baudin P, Monreal-Aguero C, Hermann G, et al. An accessible deep learning tool for voxel-wise classification of brain malignancies from perfusion MRI. *Cell Reports Medicine* (2024). doi:10.1016/j.xcrm.2024.101464.

27. Afroj M, Mondal MRH, Hassan MR, Akter S. MobDenseNet: A hybrid deep learning model for brain tumor classification using MRI. *Array* (2025). doi:10.1016/j.array.2025.100413.

28. Saeed N, Ridzuan M, Alasmawi H, Sobirov I, Yaqub M. MGMT promoter methylation status prediction using MRI scans? An extensive experimental evaluation of deep learning models. *Medical Image Analysis* (2023). doi:10.1016/j.media.2023.102989.

29. Al-Fakih A, Shazly A, Mohammed A, Elbushnaq M, Ryu K, Gu Y, et al. FLAIR MRI sequence synthesis using squeeze attention generative model for reliable brain tumor segmentation. *Alexandria Engineering Journal* **99** (2024) 108–123. doi:10.1016/j.aej.2024.05.008.

30. Ganie SM, Tanguturi RC, Alfuraydan MM. Explainable artificial intelligence-Infused hybrid transfer learning framework with multiscale feature fusion for brain tumor detection and classification. *Engineering Applications of Artificial Intelligence* (2026). doi:10.1016/j.engappai.2026.114128.

31. Nazir MI, Akter A, Wadud MAH, Uddin MA. Utilizing customized CNN for brain tumor prediction with explainable AI. *Heliyon* (2024). doi:10.1016/j.heliyon.2024.e38997.

32. Alim M, Tamim M, Sarkar S, Yousuf M, Al-Moisheer A, Alyami S, et al. Multi-teacher knowledge distillation and ensemble algorithm for efficient brain tumor classification in resource-constrained environments with explainable AI. *Knowledge-Based Systems* (2025). doi:10.1016/j.knosys.2025.114711.

33. Bhuiyan E, Khan M, Hossain S, Rahman R, Luo Q, Hossain M, et al. Classification of glioma grade and Ki-67 level prediction in MRI data: A SHAP-driven interpretation. *Computerized Medical Imaging and Graphics* (2025). doi:10.1016/j.compmedimag.2025.102578.

34. Agrawal A, Chaki J. CerebralNet meets Explainable AI: Brain tumor detection and classification with probabilistic augmentation and a deep learning approach. *Biomedical Signal Processing and Control* (2025). doi:10.1016/j.bspc.2025.108210.

35. Raju ASN, Gatla RK, Patnala PM, Reddy JGP, Prashanth A. NeuroLIME-EnsemNetX: An explainable deep ensemble intelligence for accurate brain tumor diagnosis and risk-level clinical recommendation. *Biomedical Signal Processing and Control* (2026). doi:10.1016/j.bspc.2026.109881.

36. Ayaz H, Oladimeji O, McLoughlin I, Tormey D, Booth T, Unnikrishnan S. An eXplainable deep learning model for multi-modal MRI grading of IDH-mutant astrocytomas. *Results in Engineering* (2024). doi:10.1016/j.rineng.2024.103353.

37. Gagliardi M, Maurmo D, Ruga T, Vocaturo E, Zumpano E. BrAInVision: A hybrid explainable Artificial Intelligence framework for brain MRI analysis. *Image and Vision Computing* (2025). doi:10.1016/j.imavis.2025.105629.

38. Saeed T, Khan M, Hamza A, Shabaz M, Khan W, Alhayan F, et al. Neuro-XAI: Explainable deep learning framework based on deeplabV3+ and bayesian optimization for segmentation and classification of brain tumor in MRI scans. *Journal of Neuroscience Methods* (2024). doi:10.1016/j.jneumeth.2024.110247.

39. Dang K, Vo T, Ngo L, Ha H. A deep learning framework integrating MRI image preprocessing methods for brain tumor segmentation and classification. *IBRO Neuroscience Reports* (2022). doi:10.1016/j.ibneur.2022.10.014.

40. Hasan M, Hasan M, Akter S, Mahi A, Uddin M. Enhancing brain tumor classification with a novel attention based explainable deep learning framework. *Biomedical Signal Processing and Control* (2026). doi:10.1016/j.bspc.2025.108636.

41. Ahmed S, Shil D, Souro T, Mahmood S, Khan F. Advancing brain tumor MRI classification using SwRD: A parallel swin transformer-ResNet approach. *Virtual Reality & Intelligent Hardware* (2025). doi:10.1016/j.vrih.2025.06.002.

42. Appiah R, Pulletikurthi V, Esquivel-Puentes H, Cabrera C, Hasan N, Dharmarathne S, et al. Brain tumor detection using proper orthogonal decomposition integrated with deep learning networks. *Computer Methods and Programs in Biomedicine* (2024). doi:10.1016/j.cmpb.2024.108167.

43. Al Khalil Y, Ayaz A, Lorenz C, Weese J, Pluim J, Breeuwer M. Multi-modal brain tumor segmentation via conditional synthesis with Fourier domain adaptation. *Computerized Medical Imaging and Graphics* (2024). doi:10.1016/j.compmedimag.2024.102332.

44.Yeafi A, Islam M, Yusuf MSU. A deep learning framework for 3D brain tumor segmentation and survival prediction. *Healthcare Analytics* (2025). doi:10.1016/j.health.2025.100418.

45.Rastogi A, Brugnara G, Foltyn-Dumitru M, Mahmutoglu MA, Preetha CJ, Kobler E, et al. Deep-learning-based reconstruction of undersampled MRI to reduce scan times: a multicentre, retrospective, cohort study. *The Lancet Oncology* (2024). doi:10.1016/S1470-2045(23)00641-1.

46.Guan Y, Alshammari A, Wang Y, Gul J, Imran A. ResSGA-Net: A deep learning approach for enhanced brain tumor detection and accurate classification in healthcare imaging systems. *Journal of Genetic Engineering and Biotechnology* (2026). doi:10.1016/j.jgeb.2026.100658.

47.Zhou M, Salmanpour M, Bhat S, Baur C, Menze B, Tabatabai G, et al. Generating 3D brain tumor regions in MRI using vector-quantization Generative Adversarial Networks. *Computers in Biology and Medicine* (2025). doi:10.1016/j.compbiomed.2024.109502.

48.Vidhya S, Raja PS, Sumithra R, Garuba M, Gao XZ. SEMD-Net: A transformer based approach for brain tumor segmentation and classification. *Neurocomputing* (2026). doi:10.1016/j.neucom.2026.132831.

49.Kaur A, Kukreja V, Ati M, Bansal A, Hariharan S. BrainDx: a dual-transformer framework using PVT and SegFormer for tumor diagnosis. *Biomedical Signal Processing and Control* **113** (2026) 108917. doi:10.1016/j.bspc.2025.108917.

50.Nguyen-Tat TB, Nguyen TQT, Nguyen HN, Ngo VM. Enhancing brain tumor segmentation in MRI images: A hybrid approach using UNet, attention mechanisms, and transformers. *Egyptian Informatics Journal* (2024). doi:10.1016/j.eij.2024.100528.

51.Badaoui RE, Bonmati E, Argyriou V, Villarini B. Federated learning using quality-based aggregation method for brain tumour segmentation on multimodality medical images. *Intelligent Systems with Applications* (2025). doi:10.1016/j.iswa.2025.200601.

52.Ramesh Khanna A, Thilakavathy P. Federated learning for brain tumour detection and classification using improved ShuffleNet-PCNN architecture. *Biomedical Signal Processing and Control* (2026). doi:10.1016/j.bspc.2026.109719.
