# Supplementary material for: A systematic review of machine learning approaches for MRI-based brain tumor analysis
Source: Front Comput Neurosci. 2026 Oct 12;20:1937002. doi: 10.3389/fncom.2026.1937002 (PMC13696301; doi:10.3389/fncom.2026.1937002)
Supplement: Supplementary file 1 [file Data_Sheet_1.zip › Submission_Upload/logo1-eps-converted-to.pdf]

frontiers
